# Development and validation of an instrument assessing women’s satisfaction with screening mammography in an organized breast cancer screening program

**DOI:** 10.1186/1472-6963-14-9

**Published:** 2014-01-08

**Authors:** Isabelle Bairati, Stéphane Turcotte, Geneviève Doray, France Belleau, Louise Grégoire

**Affiliations:** 1 Public Health Agency of the Capitale-Nationale, Quebec City, Canada; 2 Research Center of the CHU de Québec, Quebec City, Canada; 3 Department of surgery, faculty of medicine, Université Laval, Quebec City, Canada; 4 Québec Breast Cancer Screening Program, coordination center of the Capitale-Nationale, Quebec City, Canada

**Keywords:** Validation studies, Consumer satisfaction, Questionnaires, Mammography, Cancer screening tests

## Abstract

**Background:**

The assessment of the quality of mammography services delivered in organized breast cancer screening programs should include measures centered on women’s perceptions. The objective of this study was to develop and validate an instrument in French designed to evaluate the satisfaction of women having a screening mammography.

**Methods:**

An instrument evaluating women’s satisfaction with mammography services was developed using published research, the perceptions of screened women, the expertise of health professionals and a pilot study. Between November 9 and 21, 2011, the questionnaire was sent to 1500 consecutive women having had a screening mammography in eight radiologic facilities designated by the Québec Breast Cancer Screening Program, in Quebec City, Canada. Construct validity, convergent and discriminant validity, reliability and sensitivity of the instrument were examined.

**Results:**

A total of 819 women (55%) participated in the validation study. The factor analysis retained four satisfaction dimensions: satisfaction with 1) the technician’s skills (four items), 2) the physical environment (four items), 3) the staff’s communication skills (three items) and 4) the information given by the program (3 items). The multitrait-scaling analysis showed good convergent and discriminant validity: scaling success was 100% for all subscales. All subscales had good internal consistency (Cronbach’s alphas ≥ 0.86). The satisfaction scores were able to identify groups of women with lower levels of satisfaction, such as younger women or women with pain during breast compression.

**Conclusion:**

This brief satisfaction instrument, developed in French, showed good psychometric properties to evaluate satisfaction in women receiving mammographic services in an organized breast cancer screening program.

## Background

An organized breast cancer screening program, the Québec Breast Cancer Screening Program (PQDCS), has been implemented since 1998 in the Province of Quebec, Canada [1,2]. This publicly funded program, for women aged 50 to 69, includes a bilateral two-view screening mammography done every two years. Quality assessment of screening mammography services is primarily based on well-defined indicators of performance generated from screening and administrative databases. To complete this assessment, the public health agency of the Quebec City region has decided to add valid measures of quality of services centered on the perceptions of screened women.

Consumers’ satisfaction with health services is a measure of quality of services [3-7]. According to Donabedian [3], satisfaction consists of value judgments regarding the outcomes, process and structure of health services. Process and structure refer respectively to the activities of health professionals and the organisation of institutions, while outcome is related to a change in health status. Satisfaction with health services can also be viewed as the adequacy between the perceived quality of services and the individual’s expectations or needs (4, 7). Satisfaction with health services is consensually recognized as a multidimensional concept and must be evaluated using multi-item scales instruments [6,7].

Screening programs that effectively reduce breast cancer mortality require regular mammograms, according to guidelines. Satisfied consumers of health services are more likely to comply and persist [8,9]. In a US screening program, women satisfied with a convenient appointment time were more likely to be rescreened within 30 months [10]. In addition, satisfaction with previous mammographic experience was shown to predict future attendance [11].

Few instruments have been validated to assess satisfaction with mammography screening [12-17]. The available instruments have limitations, such as incomplete validation or insufficient psychometric properties, or they evaluate the physical and psychological experiences of women having a mammography rather than their satisfaction with the processes and events occurring during their screening mammographic experience [18]. To our knowledge, no instrument was validated for French speaking populations, such as in Quebec, Canada. The objective of this study was to develop and validate an instrument designed to evaluate the satisfaction of French speaking women having a mammography in an organized breast cancer screening program.

## Methods

### Development of the instrument

An extensive literature review was done to identify studies evaluating the satisfaction or experience of women during their mammography. All studies, using validated [12-17] or non-validated instruments for evaluating satisfaction with mammography services, were considered. Issues related to the quality of services offered to women having a mammography were extracted. Pertinent issues were also identified from other sources: 1) educational documents for the training of professionals working in the PQDCS screening centers, and 2) a qualitative study assessing the perceptions of 30 women having had a PQDCS mammography in the Quebec City area [19]. Issues were then classified according to recognized dimensions of satisfaction with health services [4-6]. We identified 39 potentially relevant issues corresponding to six dimensions of satisfaction. These issues were then discussed with health professionals working in the screening program and with women having had a PQDCS mammography.

Finally, we generated 35 items that describe the experience of women having a screening mammography. Women’s had to rate their levels of satisfaction about processes and events that were anticipated to occur for all of them. This 35 items were designed to evaluate women’s satisfaction according to six dimensions of satisfaction: 1) the staff’s communication skills, i.e. the health professionals’ ability to provide information (5 items); 2) the staff’s interpersonal skills, i.e. the way in which the health professionals interact personally with women (7 items); 3) the information provided by the program, i.e. the extent to which documents from the screening program transfer information to women (6 items); 4) the competence of the technician in charge of the mammography (7 items); 5) the accessibility/convenience of the mammography services (5 items) and 6) the physical environment, i.e. physical features of the radiologic clinics and radiologic hospital departments (5 items). A Likert scale of 10 ordered categories was chosen with only the two end-points labelled (1: extremely unsatisfied to 10: extremely satisfied).

Women’s general satisfaction was evaluated using an adaptation of the Client Satisfaction Questionnaire-3 items (CSQ score) [20]. Responses were collected using a Likert scale of 10-points (1: totally disagree to 10: totally agree). We added questions investigating socio-demographic characteristics (age, marital status, education level, employment status) and the level of pain during breast compression (from 1 (no pain) to 10 (extremely painful).

### Pre-test of the instrument

A pre-test was done among 12 consecutive women having had a mammography screening in one of the radiologic clinic designated by the PDQCS. The research assistant was instructed to 1) invite women’s having had a screening mammography to complete a questionnaire evaluating their satisfaction with their experience; 2) to inform women that the questionnaire was in development and that we needed their help to improve the understanding of the questionnaire; and 3) to ask women to report their potential difficulties for completing the questionnaire. Since the questionnaire was designed to be self-administered, no verbal instructions were given on how to fill it. Written instructions were given on the first page of the instrument. All women completed the questionnaire in about 10 minutes. Following completion of the instrument, each woman met with the research assistant and discussed her difficulties. Following this pre-test, the instructions were improved (e.g. women were asked to carefully read each item), the wording of six items was revised, and the scale for the satisfaction items was improved (addition of a happy/unhappy face above the corresponding end point).

### Field-testing of the instrument

#### Sample and data collection

Between November 9 and 21, 2011, the questionnaire was sent to 1500 consecutive women who had a PQDCS mammography in one of eight designated institutions (five clinics and three hospitals) in the Quebec City area. The questionnaire with a stamped return envelope was joined to the letter informing women of their mammography results. All questionnaires were sent within eight working days following the screening mammography and returned before January 31, 2012. Our institutional ethics committee, the *Comité d’éthique de la recherche des CSSS de la Vieille-Capitale, de Québec-Nord, de Portneuf, et de la Direction de santé publique de la Capitale-Nationale*, gave permission to conduct this survey.

### Statistical analyses

Construct validity of the satisfaction instrument was assessed using exploratory factor analysis, with a promax rotation [21,22]. The factor analysis was done among participants who had no missing data for the 35 items. Several criteria were considered to determine the number of factors: the scree test, the eigenvalue >1.00, the factor explaining at least 5% of the common variance, and the interpretability of the factors. Standardized regression coefficients of at least 0.5 were chosen to identify items with a meaningful factor loading. If an item loaded on more than one factor, then this item was not retained in subsequent analyses (factor loading > 0.40 for the second factor). When the final solution of the factor analysis was obtained, item reduction was done. An item was removed from its scale when its suppression had little effects on the Cronbach’s alpha (change of less than 1%).

The satisfaction scores were calculated for each scale, as well as the global score of satisfaction, which was based on all items. All scores varied between 1 and 10, with 10 indicating the highest level of satisfaction. Standard statistics were performed to describe the scores. Spearman correlations between satisfaction scales were generated. The same procedures were done with the CSQ-score.

A multitrait-scaling analysis was conducted to examine the convergent and discriminant validity of the instrument among all participating women. Convergent validity was supported for Spearman correlations ≥ 0.40. Using a Z test, we verified that each item had a higher correlation with its own scale (corrected for overlap) than with other scales [21]. A scaling success was counted when the item to own-scale correlation was significantly higher than the correlations of the item to other scales. Internal consistency of each scale was assessed using Cronbach’s alpha.

Concurrent validity was evaluated using the Spearman correlation between the global score of satisfaction generated from our instrument and the general score of satisfaction from the Client satisfaction Questionnaire [20]. We also examined the sensitivity of each scale of satisfaction to identify sub-groups of women. Based on the literature, we hypothesized that older women, women with lower levels of education, and those with no pain or lower levels of pain during breast compression would report higher satisfaction than the others [4,7,23-25]. Multiple linear regressions were conducted to identify women’s characteristics independently associated with each of the satisfaction scales. The statistical analyses were done with SAS, version 9.2 (SAS Institute, Cary, NC). All tests were two-sided at the level of 5%.

## Results

A total of 819 women (55%) returned their questionnaire. Of these, five questionnaires (0.6%) were excluded because of inadequate completion. Table 1 shows that 55% of the women were aged 50–59 and 41% reported higher levels of pain during breast compression (Table 1).

**Table 1:** Characteristics of the study women participating in the mammography screening program (n = 814)

**Characteristics**	**N (%)**
Age (years)	
50-54	236 (29.0)
55-59	212 (26.0)
60-64	212 (26.0)
65-69	153 (18.8)
Missing	1 (0.2)
Educational level	
University	229 (28.1)
< University	580 (71.3)
Missing	5 (0.6)
Employment status	
Employed	399 (49.0)
Unemployed	404 (49.6)
Missing	11 (1.4)
Married	
Yes	575 (70.6)
No	238 (29.2)
Missing	1 (0.2)
Pain level during compression (0–10)	
≤ 5	437 (53.7)
> 5	334 (41.0)
Missing	43 (5.3)

The factor analysis retained four factors (14 items) accounting for 100% of the common variance (Table 2). Most items had high factor loadings on only one factor and near-zero loadings for the other factors. In addition, the four factor solution corresponded to the conceptual framework adopted at the stage of questionnaire development: satisfaction with the technician’s skills (factor 1, four items), satisfaction with the physical environment (factor 2, 4 items), satisfaction with the staff’s communication skills (factor 3, three items), and satisfaction with the information given by the program (factor 4, 3 items). Among the 14 items of satisfaction, only one item had more than 4% of missing values (6.3%). This item referred to women’s satisfaction with the information concerning the possibility of having additional exams. Older women were less likely to answer this item (13% in women aged 65–69 versus 5% in others, p = 0.001). Items generated to assess satisfaction with the staff’s interpersonal skills and with the accessibility were not retained in the final solution.

**Table 2:** Factor analysis: rotated matrix of factor loadings (n = 666)

**Item number**	**Item description**	**Factors**			
		**1**	**2**	**3**	**4**
15	Staff’s explanations concerning the screening process	0.15	0.04	**0.63**	0.14
27	Information concerning the possibility of having additional exams	0.06	0.10	**0.86**	-0.16
34	Information concerning the follow-up in the screening program	-0.09	-0.04	**0.77**	0.24
12	Information concerning the advantages and disadvantages to participate in the PQDCS	0.09	-0.01	0.08	**0.65**
16	Clarity of the PQDCS informed consent form	0.04	0.05	0.21	**0.65**
5	Clarity of the information given in the PQDCS documents	-0.01	0.13	-0.10	**0.83**
14	Technician’s respectful attitude	**0.89**	-0.09	-0.04	0.09
20	Technician’s explanation	**0.77**	-0.01	0.06	0.03
26	Technician’s professionalism	**0.94**	0.03	0.04	-0.09
32	Technician’s technical competence	**0.83**	0.13	0.00	0.03
10	Cleanliness of the radiologic facilities	0.17	**0.54**	-0.02	0.21
23	Privacy allowed by the installation	0.11	**0.76**	0.01	0.03
29	Comfort of the changing room	-0.09	**0.87**	0.05	-0.05
4	Comfort of the waiting room	-0.03	**0.62**	0.06	0.16
Proportion of the common variance		74%	15%	6%	5%
Eigenvalues		30.0	6.0	2.7	2.0

Numbers in bold indicate standardized regression coefficients > 0.5.

Descriptive statistics showed high levels of satisfaction for each score (Table 3). Between 34.9% and 59.7% of women reported the highest level of satisfaction (rating of 10) for the subscales. A strong correlation of 0.68 (p < 0.001) was found between the global score of satisfaction and an independent measure of general satisfaction, the CSQ score (Cronbach’s alpha of the CSQ score = 0.90).

**Table 3:** Descriptive statistics of women’s satisfaction with the mammography screening program (n = 814)

**Satisfaction scales**	**Mean (SD)**	**Median**	**% of high response (=10)**	**Correlations**					
				**STA**	**INF**	**TEC**	**PHY**	**GLO**	**CSQ**
Staff’s communication skills (**STA**: items 15, 27, 34)	8.82 (1.53)	9.3	40.3	1.00	0.70	0.64	0.65	0.89	0.60
Information given by the program (**INF**: items 5, 12, 16)	9.34 (0.99)	10.0	52.0		1.00	0.69	0.68	0.81	0.65
Technician’s skills (**TEC**: items 14, 20, 26, 32)	9.50 (0.96)	10.0	59.7			1.00	0.62	0.76	0.71
Physical environment (**PHY**: items 4, 10, 23, 29)	9.03 (1.13)	9.5	34.9				1.00	0.88	0.59
Global satisfaction (**GLO**: all items)	9.19 (0.95)	9.5	25.3					1.00	0.68
**General satisfaction (CSQ-3 items**^**a**^**)**	9.64 (0.77)	10.0	71.7	0.60	0.65	0.71	0.59	0.68	1.00

^a^CSQ: Client Satisfaction Questionnaire from Larsen et al. [19].

The multitrait-scaling analysis showed that items correlated highly with the items from the same scales (r ≥ 0.71) and poorly with the scores of the other scales indicating a scaling success of 100% for all scales (Table 4). This demonstrated an excellent convergent and discriminant validity. For all scales, the Cronbach’s alphas were at least 0.86 showing a good internal consistency.

**Table 4:** Convergent and discriminant validity, reliability of the satisfaction instrument with the mammography screening program (n = 814)

**Dimensions**	**No. of items**	**Convergent validity (range of correlations)**	**Discriminant validity (range of correlations)**	**Scaling Success n/N**^**1**^**(%)**	**Reliability (Cronbach’s Alpha)**
Staff’s communication skills	3	0.75-0.80	0.58-0.69	9/9 (100%)	0.86
Information given by the program	3	0.75-0.78	0.59-0.67	9/9 (100%)	0.86
Technician’s skills	4	0.75-0.85	0.49-0.67	12/12 (100%)	0.93
Physical environment	4	0.71-0.74	0.52-0.66	12/12 (100%)	0.86

^1^Number of convergent correlations significantly higher than discriminant correlations/total number of correlations.

Table 5 shows that the satisfaction scores were able to differentiate groups of women. Multivariate analyses showed that older women were more satisfied than younger women for all satisfaction scales; however, this association was of borderline statistical significance for the scale assessing satisfaction with the staff’s communication skills. Satisfaction with the technician’s skills was lowest in women reporting higher levels of pain during breast compression (p < 0.001). Women’s global satisfaction was higher in older women, in those with lower levels of education and in those with the lowest levels of pain.

**Table 5:** Multivariate models of women’s characteristics on satisfaction with the mammography screening program

**Factors**	**Scales of satisfaction**									
	**Staff’s communication skills (n = 756)**		**Information given by the program (n = 752)**		**Technician’s skills (n = 758)**		**Physical environment (n = 758)**		**Global Satisfaction (n = 758)**	
	**β (SE)**	**P-value**	**β (SE)**	**P-value**	**β (SE)**	**P-value**	**β (SE)**	**P-value**	**β (SE)**	**P-value**
Age										
(60–69 y.	0.21	0.09	0.31	<0.001	0.20	0.01	0.28	0.002	0.25	0.001
vs. 50–59 y.)	(0.13)		(0.08)		(0.08)		(0.09)		(0.08)	
Education										
(≥ University	-0.41	<0.001	-0.14	0.08	-0.07	0.39	-0.11	0.20	-0.17	0.02
vs. < University)	(0.12)		(0.08)		(0.08)		(0.09)		(0.07)	
Employment										
(Employed	-0.12	0.35	0.01	0.87	0.05	0.51	-0.06	0.50	-0.03	0.74
vs. unemployed)	(0.13)		(0.08)		(0.08)		(0.09)		(0.08)	
Pain level										
(> 5 vs. ≤ 5)	-0.18	0.11	-0.12	0.09	-0.24	<0.001	-0.19	0.02	-0.19	0.005
	(0.11)		(0.07)		(0.07)		(0.08)		(0.07)	

## Discussion

This is the first study designed to develop and validate a French language instrument for assessing women’s satisfaction with a screening mammography done in the context of an organized program. This brief instrument of 14 items consists of four satisfaction subscales, which are consistent with the theoretical concepts of satisfaction (see the satisfaction questionnaire as an Additional file 1). This instrument shows good psychometric properties in terms of structure, internal reliability, and sensitivity.

Two subscales allowed women to assess their satisfaction with the process of the delivery of the screening mammography services. In this study, women’s satisfaction with the technician’s skills was identified as the most important issue. In addition, as hypothesized, this satisfaction subscale was able to identify the women having had higher levels of pain during breast compression. These results highlight the role of technicians, as perceived by women, as essential in the process of delivery of mammographic services. Organized breast cancer screening programs are well aware of this need since technologists receive regular training in screening mammography, including guidelines for breast compression [26]. Moreover, women’s satisfaction with the team’s communication skills was captured by the instrument; however, this dimension was a less important issue. This could suggest that women now have a better knowledge of the mammographic screening process and therefore need less explanation over the course of the mammography visit. Finally, none of the items designed to evaluate women’s satisfaction with the staff’s interpersonal skills was retained in the factor analysis. These items referred to the team’s respectful attitude, courtesy, availability, and ability to facilitate women’s expression and comfort. These items tended to contribute to the two previous factors; however, none loaded meaningfully to a specific factor.

Our instrument was also able to generate two subscales assessing the quality of the program structure. One novelty with this instrument was its ability to assess women’s perception regarding the quality of the documents provided by the program. In particular, items were elaborated to evaluate the quality of information in the consent form and documents explaining the advantages and disadvantages of participating in the screening program. Similarly to other validated instruments [12-14,17], our instrument also permitted to generate a measure of satisfaction with the physical environment of the radiologic facilities. The physical environment, which referred to the comfort, cleanliness, and adequacy of the installation for preserving privacy, was an important issue for screened women. Only two items measuring the satisfaction with the accessibility/convenience of the screening services loaded meaningfully to a fifth factor. This fifth factor was not retained in the final solution of the factor analysis since it explained less than 5% of the proportion of the variance.

One of the goals of this study was also to test a convenient strategy for screening programs to regularly assess women’s satisfaction, at a reasonable cost, that is not influenced by a face-to-face contact at the time of the visit. The study response rate was moderate, although comparable to other surveys using postal questionnaire without follow-up contact [7,15,27]. One possibility is that women who were unsatisfied with the screening visit may have been less prone to participate [18]. This could partly explain the high levels of satisfaction in this survey. However, this phenomenon is probably minimal since the questionnaires were anonymous and, therefore, women’s expression of dissatisfaction could not influence their future services. In addition, this hypothesis is not supported by the age distribution in the survey, which reflected well the participation rate in the PQDCS [28]. Few questionnaires were discarded (0.6%) and all items had high rates of response, except one concerning the possibility of having additional exams. In order to improve the questionnaire, we still need to evaluate whether older women, who were less prone to respond to this item, did not understand the phrasing of this item or the process of screening more broadly. Overall, the questionnaire was well accepted and answered.

Measures of satisfaction are generally recognized to be positively skewed [7]. To minimize the problem of the well-known ceiling effect in satisfaction assessment, we gave women the possibility to refine their rating for high levels of satisfaction by using a 10-point scale [21]. Despite this, a high proportion of responders gave the maximum score. As observed with the majority of the instruments evaluating consumers’ satisfaction, our instrument was able to identify women with the lowest levels of satisfaction, but it was not sensitive enough to discriminate the levels of satisfaction in satisfied women. While the ceiling effect problem could limit our ability to detect satisfaction differences between women, our instrument was able to discriminate groups of women according to recognized determinants of satisfaction, such as age, education level, and pain during breast compression [4,7,23-25]. In the context of research in health services, it would be pertinent to generate more valid estimates for better measuring the effects of factors affecting satisfaction. To reach this goal, alternative statistical methods taking into accounted the ceiling effect usually observed in the measures of satisfaction should be explored [29]. However, in the context of program evaluation, our instrument appeared to be sensitive enough to identify groups of women in which screening services could be improved.

## Conclusions

In conclusion, we developed and validated a brief instrument generating four subscales of satisfaction for evaluating the quality of mammography services in organized screening programs. This satisfaction instrument developed for French speaking women shows good psychometric properties. Additional studies should be conducted in independent population of women attending organised breast cancer screening programs to confirm the validity of this promising instrument.

## Competing interests

The authors declare that they have no competing interest.

## Authors’ contributions

All authors have made substantial contributions for the conception and design (in particular IB and LG), the acquisition of data (in particular GD and FB) the analysis (in particular SD) and the interpretation (all authors). IB was responsible for the writing of the article. All authors have revised the drafts. They all gave their final approval of the version to be published.

## Pre-publication history

The pre-publication history for this paper can be accessed here:

http://www.biomedcentral.com/1472-6963/14/9/prepub

## Supplementary Material

Additional file 1 The satisfaction questionnaire is added as an additional file. Click here for file
