# Supplementary material for: Development and validation of an instrument assessing women’s satisfaction with screening mammography in an organized breast cancer screening program
Source: BMC Health Serv Res. 2014 Jan 8;14:9. doi: 10.1186/1472-6963-14-9 (PMC3893508; doi:10.1186/1472-6963-14-9)
Supplement: Additional file 1 — The satisfaction questionnaire is added as an additional file. [file 1472-6963-14-9-S1.pdf]

Madame,

Nous désirons connaître votre satisfaction concernant les services reçus lors de votre mammographie de dépistage.

Votre opinion est très importante pour nous. Chaque énoncé du questionnaire reflète la qualité des services que vous devriez avoir reçus. Nous vous demandons de **lire attentivement tous les énoncés** et de **répondre à chacun d'eux.**

Pour chaque énoncé, vous devez mettre une note sur 10 :

- 1 signifiant que vous êtes extrêmement insatisfaite;
- 10 signifiant que vous êtes extrêmement satisfaite.

Cochez votre réponse

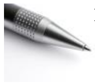

√

Niveau de satisfaction											
Extrêmement insatisfaite	→										Extrêmement satisfaite
☹	**1** ①	**2** ②	**3** ③	**4** ④	**5** ⑤	**6** ⑥	**7** ⑦	**8** ⑧	**9** ⑨	**10** ⑩	☺

Nous vous remercions de votre collaboration.

Définition des mots utilisés dans le questionnaire

Équipe : Ensemble du personnel du centre de dépistage (personnel à l'accueil et technologue)

Centre de dépistage : Endroit où vous avez passé votre mammographie de dépistage (clinique de radiologie ou hôpital)

Mammographie : Dernière radiographie de dépistage des seins que vous venez de passer

Votre satisfaction nous importe!

Votre satisfaction nous importe!

SUR UNE ÉCHELLE DE 1 À 10,
COMMENT NOTERIEZ-VOUS VOTRE NIVEAU DE SATISFACTION ?

	Niveau de satisfaction									
	Extrêmement insatisfaite →									Extrêmement satisfaite
	☹ 1	2	3	4	5	6	7	8	9	10 ☺
Le confort de la salle d'attente du centre de dépistage	①	②	③	④	⑤	⑥	⑦	⑧	⑨	⑩
La clarté de l'information donnée dans les documents du programme de dépistage	①	②	③	④	⑤	⑥	⑦	⑧	⑨	⑩
La propreté des locaux du centre de dépistage	①	②	③	④	⑤	⑥	⑦	⑧	⑨	⑩
L'information donnée dans les documents du programme de dépistage sur les avantages et inconvénients à participer au programme	①	②	③	④	⑤	⑥	⑦	⑧	⑨	⑩
L'attitude professionnelle de la technologue lorsqu'elle a placé vos seins sur le mammographe	①	②	③	④	⑤	⑥	⑦	⑧	⑨	⑩
Les explications données par l'équipe sur ce qui se passe avant, pendant et après votre visite au centre de dépistage	①	②	③	④	⑤	⑥	⑦	⑧	⑨	⑩
La clarté du formulaire à signer pour accepter de participer au programme de dépistage	①	②	③	④	⑤	⑥	⑦	⑧	⑨	⑩
Le professionnalisme de la technologue à vous avertir à quel moment débutait la compression du sein	①	②	③	④	⑤	⑥	⑦	⑧	⑨	⑩
L'aménagement des locaux du centre de dépistage qui permet de préserver l'intimité	①	②	③	④	⑤	⑥	⑦	⑧	⑨	⑩
Le professionnalisme de la technologue à réaliser la compression du sein de manière progressive pour limiter votre inconfort	①	②	③	④	⑤	⑥	⑦	⑧	⑨	⑩
L'information donnée par l'équipe sur la possibilité d'avoir des examens additionnels après la mammographie	①	②	③	④	⑤	⑥	⑦	⑧	⑨	⑩
Le confort de la cabine de déshabillage du centre de dépistage	①	②	③	④	⑤	⑥	⑦	⑧	⑨	⑩
Les ajustements techniques faits par la technologue pour favoriser votre confort au moment de la mammographie	①	②	③	④	⑤	⑥	⑦	⑧	⑨	⑩
L'information donnée sur le suivi offert par le programme de dépistage	①	②	③	④	⑤	⑥	⑦	⑧	⑨	⑩

© Isabelle Bairati, 2014. Direction régionale de santé publique de la Capitale-Nationale, Centre de recherche du CHU de Québec et Université Laval
